# Patterns of Cannabis and Alcohol Co-Use: Substitution Versus Complementary Effects

**DOI:** 10.35946/arcr.v42.1.04

**Published:** 2022-02-10

**Authors:** Rachel L. Gunn, Elizabeth R. Aston, Jane Metrik

**Affiliations:** 1 Center for Alcohol and Addiction Studies, Brown University School of Public Health, Providence, Rhode Island; 2 Providence VA Medical Center, Providence, Rhode Island

**Keywords:** alcohol, cannabis, marijuana, concurrent use, co-use

## Abstract

**PURPOSE:**

The purpose of this review is to discuss the literature regarding the concurrent use (co-use) of alcohol and cannabis and competing hypotheses as to whether cannabis acts as a substitute for (i.e., replacing the effects of alcohol, resulting in decreased use) or a complement to (i.e., used to enhance the effects of alcohol, resulting in increased use) alcohol. The impact of cannabis use on alcohol-related outcomes has received increased attention in the wake of ongoing legalization of cannabis for both medical and recreational purposes. Evidence for both hypotheses exists in the literature across a broad range of data collection methods and samples and is carefully reviewed here. In addition, various mechanisms by which cannabis may act as an alcohol substitute or complement are explored in depth with the goal of better understanding equivocal findings.

**SEARCH METHODS:**

This review includes articles that were identified from a search for studies on alcohol and cannabis co-use, with a specific focus on studies exploring complementary versus substitution aspects of co-use. Search terms were included in Google Scholar, PsycINFO, MEDLINE, and Web of Science. Eligible studies were those that measured alcohol and cannabis co-use in human samples in laboratory, survey, or ecological momentary assessment studies, or that directly referenced substitution or complementary patterns of use.

**SEARCH RESULTS:**

Search results returned 650 articles, with 95 meeting inclusion criteria.

**DISCUSSION AND CONCLUSIONS:**

Results of this review reveal compelling evidence for both substitution and complementary effects, suggesting nuanced yet significant distinctions across different populations examined in these studies. Several mechanisms for the impact of cannabis use on alcohol-related outcomes are identified, including patterns and context of co-use, timing and order of use, cannabinoid formulation, pharmacokinetic interactions, and user characteristics (including diagnostic status), all of which may influence substitution versus complementary effects. This review will inform future research studies examining this topic in both clinical and community samples and aid in the development of treatment and prevention efforts targeting those populations most vulnerable to negative consequences of co-use. Finally, this review highlights the need for additional research in more diverse samples and the use of mixed-methods designs to examine both pharmacological and contextual influences on co-use.

Use of alcohol and related problems cause significant global and individual health-related harms, and alcohol use is currently the third-leading cause of preventable death in the United States.^1^,^2^ Alcohol and cannabis are among the most commonly used psychoactive substances in the United States.^3^ Although concurrent use of alcohol and cannabis (i.e., co-use: defined as using both substances, but not necessarily so that their effects overlap) has been linked to increased alcohol consumption and alcohol-related consequences compared to single substance use,^4^–^6^ findings as to whether cannabis use contributes to or reduces alcohol-related harms are mixed. In particular, reviews of this topic have identified competing theories, namely whether cannabis acts as a substitute (i.e., replacing the effects of alcohol, resulting in decreased use) or a complement (i.e., used to enhance the effects of alcohol, resulting in increased use).^7^–^9^ Here, the current literature is reviewed, and potential mechanisms whereby cannabis use is associated with alcohol-related behaviors and their substitution versus complementary effects are discussed. There is also a relevant distinction between concurrent use (i.e., co-use) and simultaneous use (i.e., using both substances together so that the effects overlap), which is linked to a unique set of consequences in adult and adolescent samples.^6^,^10^ The current review focuses on co-use generally and the impact of cannabis use specifically on alcohol use and related outcomes, whereas the topic of simultaneous use is reviewed elsewhere in this topic series.^11^

## Search Methods

A literature search was conducted to identify articles for this review via Google Scholar, PsycINFO, MEDLINE, and Web of Science to identify studies that examined whether cannabis acts as a substitute for or complement to alcohol use and related outcomes. Search terms used were (1) alcohol, (2) cannabis, mari*uana, (3) co-use, concurrent use, (4) substitut*, and (5) complement*. Articles were eligible for this review if they examined both cannabis and alcohol use in the same human sample, or if they directly referenced substitution or complementary hypotheses of alcohol and cannabis co-use. In addition to results of these searches, citations were identified within articles as relevant. Finally, colleagues and experts in this area were contacted to inquire about relevant work that was under review or in press.

## Results

Search results yielded more than 650 articles, with 95 articles meeting inclusion criteria. The results of this search are organized based on sample composition—clinical or treatment-seeking or -engaged and non–treatment-seeking samples—as motivation for substance use and several other clinical characteristics have been shown to differ between these two subgroups.^12^,^13^ Following this, the review discusses potential mechanisms of the effects of co-use, including pharmacological and behavioral effects of combined alcohol and cannabis use, patterns of co-use, individual differences and user characteristics that may impact co-use, and neurobiological systems that may play a role.

### Sample Composition

#### Treatment-seeking or -engaged samples

In addition to the prospective association between cannabis use and development and persistence of alcohol use disorder (AUD),^14^,^15^ there is evidence that cannabis use may be detrimental to AUD treatment. For instance, cannabis use after discharge from inpatient AUD treatment has been associated with resumed alcohol use.^16^ In another study of individuals with AUD recruited during a randomized controlled trial for chronic disease management, cannabis use at study entry was prospectively associated with reduced odds of abstinence from alcohol 1 year post-treatment.^17^ Secondary analyses of the Combined Pharmacotherapies and Behavioral Interventions (COMBINE) Study data were conducted to examine the effects of cannabis use on drinking and on alcohol-related consequences 1 year posttreatment.^18^,^19^ In support of the complementary hypothesis, any cannabis use relative to nonuse during treatment was associated with fewer abstinent days at the end of treatment. However, a nuanced association was uncovered such that those who used cannabis once or twice per month had significantly fewer alcohol-abstinent days after treatment. Contrary to the complementary hypothesis, those who used cannabis more frequently (more than twice per month) did not report fewer days abstinent from alcohol. Similarly, those who used cannabis very infrequently (less than once per month) also did not differ from abstainers in terms of their alcohol treatment outcomes. More frequent cannabis use during AUD treatment in Project COMBINE also was associated with increased alcohol-related physical consequences 1 year after treatment.^19^ Similarly, another secondary analysis of the U.S. National Alcohol Survey data from the general population subsamples of individuals previously treated for AUD showed that mid-level use (use of cannabis more than monthly but less than weekly) was associated with drinking more frequently, having more drinks per drinking occasion, and being more likely to experience alcohol-related harms relative to abstainers.^20^ Together, these studies suggest that cannabis use may be complementary to alcohol use among individuals who have received treatment for AUD, although perhaps only at certain frequencies of use.^18^,^20^

Conversely, there is some evidence of complementarity among those who drink heavily or those with AUD such that reductions in cannabis use are associated with reduced alcohol use. For example, reduction in cannabis use after treatment for cannabis use disorder (CUD) was associated with concurrent reduction in alcohol use among those with AUD diagnosis.^21^ Concomitant reductions in cannabis use and alcohol use were similarly observed among those who used cannabis, drank heavily (for men, > 14 drinks per week or ≥ 5 drinks per occasion at least once per month over the past 12 months; for women, > 7 drinks per week or ≥ 4 drinks per occasion at least once per month), and were engaged in alcohol interventions.^22^,^23^

In contrast to these findings suggesting complementary use, patients with AUD report using cannabis specifically to reduce drinking and find it to be an effective substitute for alcohol.^24^ In a recent study, cannabis use was assessed during a randomized controlled trial for AUD among enrollees characterized as “heavy drinkers” (defined as 14 drinks/week on average during the past 3 months). Number of cannabis use days (versus days when cannabis was not used) during treatment was associated with reduced alcohol consumption in both frequent and infrequent cannabis users.^25^ Conversely, among adolescents undergoing a contingency management intervention for cannabis, an increase in drinking was observed when participants were not using cannabis, whereas a reduction in drinking was observed after cannabis use was reinitiated.^26^ This inverse association between cannabis use and alcohol use while in treatment suggests that cannabis in fact may function effectively for some individuals as a substitute for alcohol, but may serve as a complement for others, and thus increasing drinking or exacerbating other alcohol treatment outcomes. Individual differences may be important factors in whether alcohol acts as a substitute for or a complement to cannabis use. These factors and other mechanisms of action are discussed further below.

#### Non-treatment samples

Studies of co-use associations among individuals not engaged in treatment also help improve our understanding of whether cannabis use leads to increased (i.e., complementary) or decreased (i.e., substitution) drinking. Calendar-assisted interview data in a sample of returning veterans who reported being more likely to drink and drink heavily (> 5 drinks for men/4 drinks for women) on days when they used cannabis suggest a potential complementary pattern.^27^ A similar pattern was also found in college students who were interviewed weekly across the first 2 years of college.^28^ In another study of college students, complementary consumption was also found in the overall sample.^29^ However, students who were more likely to use substances to cope were less likely to use cannabis on evenings when they also drank, suggesting this subpopulation may be more likely to engage in substitution.^29^ Studies that specifically examined simultaneous use also found that simultaneous use in non–treatment-seeking users was associated with a heavier quantity of drinking than concurrent use (i.e., using both substances but not at the same time)^6^,^10^,^30^ or alcohol use alone.^31^ This may suggest that the two drugs are more likely to act as complements when they are used in closer proximity to enhance psychoactive effects. As noted above, a more comprehensive review of the impact of simultaneous use on alcohol outcomes is available elsewhere in the topic series.^11^

Research examining the impact of co-use on various substance-related consequences also provides evidence for complementary effects of these two drugs. In college student and young adult samples, significant associations between cannabis use and alcohol-related consequences have been found at the weekly^28^ and daily^32^,^33^ levels. Frequent co-use also has been linked to behavioral problems in emergency room samples.^5^ Among college students who reported moderate drinking compared with the rest of the sample (measured as recent quantity and frequency of drinking), cannabis use was associated with more alcohol-related problems compared to students who drink at similar levels but do not use cannabis.^34^ At the neurocognitive level, co-use during adolescence also has been linked to unique neurocognitive abnormalities compared to single substance use.^35^ Longitudinal evidence for complementarity in consequences also exists among adult cannabis users, suggesting that those who continue to use cannabis (compared to abstainers) experience more alcohol-related problems.^36^ However, several recent studies suggest that alcohol consumption (i.e., quantity of alcohol consumed at the event level) is a stronger predictor of negative consequences compared to co-use.^31^,^37^–^40^ The complexity of these findings is discussed in more detail below in the section on patterns of co-use.

In contrast to these studies suggesting complementary patterns, studies that examined the impact of cannabis abstinence on drinking found that abstinence is associated with increased drinking or craving for alcohol in non–treatment-seeking cannabis users.^7^,^8^,^41^,^42^ In further support of the substitution hypothesis, findings from a recent within-subjects, placebo-controlled, laboratory study of a non–treatment-seeking sample of persons who used both alcohol and cannabis also suggested that administration of delta-9-tetrahydrocannabinol (THC; 3% and 7%) in smoked cannabis acutely reduced the amount of alcohol consumed on a subsequent drinking task in which participants chose either to drink their preferred alcoholic beverage or receive monetary reinforcement for drinks not consumed.^43^ This study also found that THC acutely reduced some dimensions of alcohol craving and alcohol demand measured prior to the alcohol choice task. This direct test of the effect of cannabis on alcohol use in the laboratory suggests that cannabis use prior to the onset of drinking may increase the likelihood that substitution occurs at the event level. Additional consideration of the importance of the order in which substances are consumed and patterns of use is discussed below. Interestingly, in surveys of consumers of medical cannabis, respondents directly endorsed use of cannabis as a substitute for alcohol.^24^,^44^–^46^ Although it is not clear whether these findings translate to long-term longitudinal research in drinking, the use of medical cannabis as a substitute for alcohol is an important area for future longitudinal research in both treatment-seeking and non–treatment-seeking populations. A more nuanced discussion of medical (versus recreational) cannabis use in relation to alcohol use follows below.

#### Epidemiology and policy research

Initial evidence for substitution comes from epidemiology and policy-level research. In 2014, Anderson and Rees predicted that cannabis legalization would lead to increased cannabis use, reduced alcohol use, and reduced social harms associated with alcohol.^47^ In addition, there has been evidence suggesting increased (almost double) emergency department visits involving alcohol and cannabis exposure among youth.^48^,^49^ However, a recent review of policy literature suggests that medical cannabis laws and other cannabis-related policies resulted in reduction in alcohol sales and alcohol-related fatalities after legalization of cannabis (i.e., substitution), with fewer studies supporting complementary effects or neutral or inconclusive evidence.^9^ Of note, examination of complementary versus substitution evidence at a policy level requires a more thorough evaluation of specific laws and is beyond the scope of this review. A more comprehensive review of policy-level work is undertaken elsewhere in this topic series.^50^

#### Summary

Evidence exists for both substitution and complementary effects across treatment-engaged and community populations of people who co-use alcohol and cannabis; however, individual differences other than treatment engagement (e.g., frequency of use) may contribute to mixed findings within and between these groups. It should be noted that some studies suggest neither substitution nor complementary effects. For example, studies found no change in drinking for samples who are engaged in CUD treatment^51^,^52^ and for a non–treatment-seeking sample that had reduced or abstained from cannabis use after having used it daily.^53^ Studies that examine nuanced associations are reviewed in the next section on mechanisms that may aid in understanding the effects of cannabis on alcohol outcomes.

### Mechanisms of the Effects of Co-Use

#### Pharmacological and behavioral effects of co-use

Cannabis has been shown to enhance the positive (i.e., pleasurable) effects of alcohol, increase subjective intoxication, and increase blood alcohol levels at various doses.^54^–^56^ For example, smoking low-potency cannabis (2.5% THC) paired with consumption of a low alcohol dose (0.35 g/kg) increased the number of positive subjective effects endorsed, as well as their duration, and led to higher plasma levels of THC (compared to placebo). In contrast, THC of similar potency paired with a higher dose of alcohol (0.7 g/kg) dampened the increase in plasma ethanol levels and reduced the number and duration of positive subjective effects, even though THC plasma levels were higher than for any other combination of THC potency and alcohol dose.^54^,^55^ Collectively, these findings suggest that the combination of cannabis and alcohol may be more reinforcing at lower alcohol doses than at higher doses. The few human laboratory studies examining the coadministration of alcohol and cannabis also highlight the synergistic effects of the two drugs (i.e., pharmacokinetic interactions).^54^–^57^ In other words, there is consistent evidence that the coadministration of alcohol and cannabis results in increased impairment on a number of behavioral and neurocognitive measures compared to single use of either substance.^58^–^64^ For example, in the presence of alcohol, significantly higher levels of blood THC and 11-hydroxy-delta-9-tetrahydrocannabinol (11-OH-THC, the main active metabolite of THC) are detected, which may explicate increased impairment typically observed following cannabis and alcohol coadministration.^56^ However, these studies present limited data regarding the acute influence of cannabis on motivation to consume alcohol. To date, the only study that has directly tested acute effects of cannabis (7% and 3% THC) on alcohol consumption found that smoked cannabis, relative to placebo, acutely reduced the amount of alcohol consumed on a subsequent drinking task.^43^ This finding is in line with another laboratory study examining the effects of combined cannabis and alcohol on craving—THC (2.5 mg) with a low dose of alcohol (0.2 g/kg) was actually found to attenuate self-reported “want more drug” (compared to alcohol alone) in a sample of healthy adult volunteers.^57^

Similar to laboratory findings, persons who report regular use of alcohol and cannabis in the natural environment also report an increase in the intoxicating effects of cannabis on days when alcohol is also used.^65^ Reports of young adults who engage in simultaneous use suggest that they endorse “cross-fading” motives for using the substances (i.e., using the substances together to achieve increased intoxication).^66^ However, what is less clear is whether this increased intoxication consistently motivates increased use in the moment, or whether there are individual differences that account for this association.

#### Patterns of co-use

The order in which substances are used (i.e., cannabis or alcohol first) has been shown to predict alcohol consumption at the daily level in a sample of college students who use alcohol and cannabis. Specifically, students reported drinking less on days when they reported using cannabis first.^39^ This work also controlled for between-person differences, suggesting this effect of order cannot be attributed to person-level differences in cannabis use. However, order of substance use did not predict alcohol-related consequences. This work—in addition to that conducted by others^31^,^37^,^38^,^40^—suggests there are clinically meaningful distinctions in the prediction of alcohol use versus alcohol-related problems. Even when cannabis effects are accounted for in the statistical models, alcohol quantity appears to largely drive the association between co-use (or simultaneous use) and alcohol-related consequences. In other words, the number of drinks consumed in a day or during a drinking event seems to be a more robust predictor of consequences experienced than order of substance use or co-use versus alcohol use only. However, given that prior work has suggested that co-use may lead to increased alcohol consumption, the association between co-use and consequences may be indirectly driven by number of drinks consumed. Together, these studies underscore the importance of considering all aspects of co-use patterns, including which substance was initiated first in a co-use event, the amount of each drug used, mode or formulation, and perhaps the duration of each use occasion.

Context of co-use is another characteristic that may impact alcohol-related outcomes. A large body of literature has examined the influence of sociocontextual factors on substance use generally,^67^ but significantly less work has focused on co-use of alcohol and cannabis specifically. However, recent work has found that social context (i.e., being with others versus being alone) predicts co-use compared to single substance use in both adolescent and young adult samples.^37^,^68^,^69^ Location of use also predicts co-use over single substance use, in that simultaneous use is more likely to occur at a friend’s place, at a party, and when people are around.^69^ More work is needed to examine how complex contextual (e.g., social, location, situational) factors interact to predict co-use in more diverse samples, and in treatment-seeking samples, as this may help to inform intervention and prevention efforts.

#### Individual differences and user characteristics

Between-person differences (i.e., user characteristics) are essential to consider as moderators of the impact of co-use or cannabis use on alcohol-related outcomes. One particularly prominent user characteristic is the use of cannabis for medical or recreational reasons. The 2017 National Academy of Sciences review reported beneficial effects of cannabinoids for several medical conditions, including chronic pain, nausea, and muscle spasms.^70^ Aside from evidence for symptom relief in medical conditions such as neuropathic pain and multiple sclerosis, evidence regarding therapeutic effects of cannabis for many other conditions remains elusive. Despite this insufficient evidence, the majority of states have legalized cannabis for medical use, and a number of individuals report using cannabis for medical purposes or assume cannabis has potential health benefits for a variety of physical and mental health conditions.

Although the distinction between “medical” and “recreational” use is likely a false dichotomy, particularly at the between-person level,^71^ as individuals may use cannabis for a variety of reasons, several studies have sought to understand how traditional “medical” uses of cannabis may have a unique impact on alcohol-related outcomes. A recent review of the topic found that studies that specifically recruited “medical users” tended to support the substitution hypothesis.^9^ For example, in a study of patients using cannabis for medical reasons in California, 40% of the sample directly reported using cannabis as a substitute for alcohol.^24^ This intentional substitution also has been reported among veterans who endorsed using cannabis for medicinal reasons^44^ and patients registered to use medical cannabis in Canada.^45^,^46^ In line with this, studies that compare “medicinal” users to nonmedical or “recreational” users find that those who use medical cannabis drink less.^72^–^75^ For instance, in studies of returning veterans, less alcohol was consumed among those who reported having used cannabis for medical reasons as compared to those who reported only recreational cannabis use.^76^ Specifically, those who used medical cannabis reported consuming less alcohol on days when cannabis was used, but those who used recreational cannabis reported drinking more on cannabis use days. Further, veterans using cannabis for medical reasons who directly endorsed alcohol substitution motives for cannabis use were more likely to drink less on cannabis use days.^44^ In another survey on co-use, those using cannabis to treat a medical condition reported drinking less often and having fewer drinks per drinking occasion compared to those who endorsed cannabis use for reasons other than a medical condition.^77^ Finally, a large population-based study examined whether having a medical cannabis recommendation from a practitioner had any effect on alcohol consumption among participants who used both alcohol and cannabis. Those with a medical cannabis recommendation showed lower quantity and frequency of alcohol consumption and had lower scores (i.e., alcohol-related problems) on the Alcohol Use Disorders Identification Test (AUDIT) compared to cannabis users without a medical recommendation.^78^ Together, this work suggests medical use of cannabis, although difficult to define and not likely to be easily distinguished from “recreational” use,^71^ may be associated with less alcohol use and fewer alcohol-related problems.

Age may be another user characteristic that impacts whether cannabis acts as a substitute for or a complement to alcohol and should be considered given recent increases in cannabis use among specific age groups, such as young adults and adults over age 50.^79^ Very little work has examined the role of age on co-use. One study examined the impact of medical cannabis laws by age (comparing adolescents to young adults) and found increases in binge drinking in states with medical cannabis laws, but only among those age 21 and older, not among those ages 12 to 20.^80^ Although this study is preliminary and correlational in nature, the researchers also found that cannabis use increased in this group after legalization, suggesting that young adults may be more susceptible than adolescents to the complementary effects of cannabis on alcohol use. An alternative consideration may be that adolescents in most states are unable to obtain a medical cannabis card until age 18, which complicates the ability to disentangle potential complementary effects from age-related restrictions to accessing cannabis. In line with this, the review by Risso and colleagues also found that, overall, studies of young adults were more likely to show complementary patterns of consumption (versus substitution patterns).^9^ Although preliminary work has begun to examine the potential role of age on co-use, future research should continue to investigate the effects of cannabis and varying cannabinoid compositions on alcohol consumption and patterns of use among diverse age samples.

Several recent investigations have assessed the influence of sex as a moderator of alcohol and cannabis co-use and have reported mixed findings. Much work suggests that the prevalence of co-use is substantially higher among males than females.^6^,^30^,^81^,^82^ Purcell and colleagues found that males were more likely to co-use alcohol, tobacco, and cannabis, but females were less likely to use all three substances.^83^ However, sex differences were no longer significant when controlling for socioeconomic status; overall, males and females were equally likely to co-use these substances. Roche and colleagues studied event-level reports of alcohol, tobacco, and cannabis use occurring on the same day among individuals endorsing alcohol use.^84^ Sex was found to moderate certain patterns of co- or tri-substance use; namely, the association between alcohol and cannabis co-use was greater in males. Moreover, there was an additive effect of co-use of alcohol with tobacco and of cannabis with tobacco on odds of same-day tri-use of tobacco, cannabis, and alcohol, and this effect was more robust in females. Wright and colleagues conducted a laboratory drug administration study to assess for the presence of sex differences in the acute pharmacological effects of alcohol and cannabis co-use.^85^ Alcohol and cannabis were administered concurrently in the laboratory using fixed-dose (target, .08% blood alcohol concentration measured through breath) and ad libitum (12.5% THC cannabis) procedures. When alcohol and cannabis were co-administered, females smoked less cannabis as compared to males. Despite this, there were no effects of sex on blood THC concentration, blood pressure, self-reported subjective drug effects, or cognitive assessments. Thus, females were found to experience similar pharmacological and subjective effects of co-use as males, despite differential titration of cannabis in the ad libitum paradigm. Another laboratory drug administration study, conducted by Venegas and colleagues, suggested that administration of alcohol increased craving for cannabis in males but not females;^86^ thus, craving may be a mechanism by which alcohol increases risk of co-use in males specifically. Collectively, this research on the influence of sex on co-use suggests that sex appears to influence patterns of co-use, drug self-administration, and craving. Subsequent work should continue to probe the impact of sex on co-use, as well as the impact of gender, for which there is a paucity of work.

Finally, diagnostic status—or the degree of problematic alcohol or cannabis use—is an essential factor to consider when understanding the impact of cannabis use on alcohol consumption and related problems. In particular, in a recent study of veterans who used both alcohol and cannabis, a calendar-assisted data collection method indicated that daily cannabis use was associated with more alcohol consumption among individuals with AUD or both AUD and CUD, but not those with CUD alone. In fact, those with CUD reported drinking less on cannabis use days compared to non-cannabis use days.^27^ In another study of college students, higher AUDIT scores before entering college predicted heavier alcohol use on cannabis use days versus nonuse days and more negative alcohol consequences on weeks when more cannabis was used.^28^

#### The endocannabinoid system and alcohol

The endocannabinoid system regulates both cannabis and alcohol reinforcement, effectively motivating and influencing use of both substances.^87^–^89^ Although it is beyond the scope of this review to discuss the endocannabinoid system in depth (addressed elsewhere in this topic series^90^,^91^), preclinical models show that cannabinoid receptor agonists and antagonists stimulate and suppress the motivational aspects of alcohol, including its consumption and self-administration.^87^ Moreover, long-term exposure to alcohol has been shown to contribute to disruption in endocannabinoid signaling.^58^,^92^ Specifically, chronic alcohol consumption leads to elevated levels of endogenous cannabinoids, ultimately facilitating the downregulation of the cannabinoid receptor type 1,^89^,^93^ a G-coupled receptor that facilitates the psychoactive, intoxicating, and rewarding or positive effects of cannabis.^94^,^95^ This work, taken together, supports the existence of cross-tolerance between alcohol and certain cannabinoids, both exogenous and endogenous. This cross-tolerance could be interpreted to lead to complementary or substitution effects, in that users may seek to use both to increase desired effects of the drug or may effectively substitute one drug for the other in the event they are attempting to reduce their use of a single substance.

#### Specific cannabinoids and alcohol use and related outcomes

The complexity of cannabinoid composition (e.g., THC-dominant versus cannabidiol [CBD]-dominant cannabis strains), cannabis use patterns (e.g., frequency of use), and formulations (e.g., flower, concentrates, edibles) warrants the investigation of a number of cannabis-specific factors that may moderate the impact of co-use on alcohol outcomes.^96^ Prominent among these are cannabinoid composition, potency, and formulation and their pharmacokinetic and pharmacodynamic effects. Although hundreds of cannabinoids in the cannabis plant have been isolated, the two most used and studied are THC (psychoactive) and CBD (nonpsychoactive). In contrast to THC, the nonpsychoactive properties of CBD and its reduced classification as a Schedule I drug in the U.S. Controlled Substances Act have resulted in an influx in the production and consumption of CBD-based products. These products are used as a natural remedy for a wide variety of health issues because of the potential for antioxidant, anti-inflammatory, and analgesic effects.

In addition to this widespread commercial use of CBD, recent preclinical and clinical evidence suggests that CBD may show some efficacy in treatment of a variety of conditions.^97^ Among these is the treatment of alcohol-related problems and AUD.^98^,^99^ Preclinical animal models suggest that CBD dampens preference for alcohol, alcohol seeking,^100^–^102^ and alcohol-related functional harms, such as those to the liver and brain.^103^–^106^ There is significantly less work examining the effects of CBD on alcohol use in humans. In one survey of persons who used cannabis and also drank alcohol, those who reported using products with a higher THC-to-CBD ratio also reported drinking less on drinking days.^77^ In a second quasi-experimental study of persons who used cannabis and alcohol, those assigned to purchase and consume CBD products ad libitum, compared to THC or CBD+THC products, reported fewer drinking days and consumed fewer drinks on drinking days.^107^ However, there were no differences on either outcome in the groups that were assigned THC compared to THC+CBD, suggesting that CBD does not attenuate the effects of THC on drinking frequency or quantity.^107^ This study, although preliminary and not placebo controlled, suggests that use of cannabis products primarily containing CBD is associated with less drinking than use of products containing THC or CBD+THC. This reduction in drinking may be explained by the therapeutic potential of the endocannabinoid system in reducing negative affect among those with AUD or alcohol-related problems.^108^ The endocannabinoid system shows promise as a potential target for pharmacological treatments for both AUD and CUD via various mechanisms. Preclinical work suggests that certain ligands that inhibit degradation of endogenous cannabinoids are promising pharmacotherapy targets for both AUD and CUD treatment.^109^–^112^ Cannabinoids also have been shown to reduce the likelihood of development of AUD via their impact on the gastrointestinal and immune system.^113^ However, this research is in its infancy, and several ongoing clinical trials seek to better understand the potential of CBD to improve AUD symptoms (i.e., NCT03248167; NCT03252756).

In addition to cannabinoid content, recent work suggests that THC content (i.e., potency) has a significant impact on alcohol-related outcomes. For instance, the use of high-potency products, such as cannabis concentrates, was associated with more alcohol consequences on co-use days among college students who use both alcohol and cannabis.^114^ This finding has been replicated in another study based on online surveys of respondents who reported co-use of alcohol and cannabis; respondents were categorized by high- versus low-THC product use. Those categorized as using high-THC products reported drinking more on cannabis use days relative to those who used low-THC products.^77^ The significant variability in cannabinoid content and potency in cannabis products, paired with this preliminary evidence that cannabinoid content is associated with alcohol consumption, calls for more controlled research on the impact of various cannabinoids on alcohol use and alcohol-related outcomes (e.g., craving, consequences, high-intensity drinking).

## Conclusions and Recommendations for Future Work

**Figure 1:**
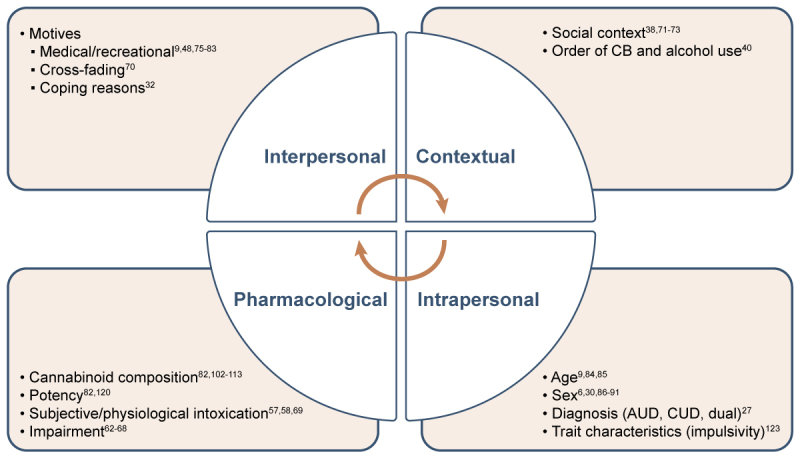
Mechanisms of the effects of cannabis and alcohol co-use *Note:* AUD, alcohol use disorder; CB, cannabis; CUD, cannabis use disorder.

Reviews and discussions on cannabis and alcohol co-use thus far have highlighted a mixed set of results regarding whether cannabis acts as a substitute or a complement to alcohol.^7^,^9^,^48^,^115^ Although this review is similarly inconclusive given there is evidence for both hypotheses (see Table 1 for summary), several additional moderators and potential mechanisms that help elucidate this complex question and pave the way for future research into this timely topic also have been highlighted (see Figure 1 for summary). Although this review is organized by studies of treatment-seeking or treatment-engaged samples versus those that are not, treatment status itself does not seem to be a clear moderator or indicator of whether cannabis acts as a substitute or complement to alcohol. However, several other mechanisms were identified. For instance, cannabis formulations, including specific cannabinoids (i.e., THC versus CBD), and potency may play a role in whether cannabis acts as a substitute for or a complement to alcohol use, as more compelling preliminary evidence exists that CBD (versus THC) may act as a substitute for alcohol.^98^,^99^,^107^,^113^ This evidence is preliminary, however, and additional research and results from ongoing clinical trials are needed to draw definitive conclusions regarding the therapeutic potential of CBD in the treatment of AUD and alcohol-related problems.

In addition, there seems to be a sample-dependent distinction between the impact of cannabis use on alcohol consumption and alcohol-related problems. For instance, evidence among non–treatment-seeking adolescents, young adults, and college students who co-use cannabis and alcohol suggests cannabis may act as a complement to alcohol, given that more drinking is often observed during co-use days or events (and, in particular, simultaneous use events), compared to single substance use.^6^,^10^,^28^,^30^,^31^,^116^ However, it seems that more frequent or problematic use of a single substance (i.e., alcohol or cannabis) among people who use both also may be indicative of whether cannabis acts as a substitute or complement. For instance, several studies suggest that cannabis use may lead to drinking among those in treatment for AUD (i.e., complementary use).^16^–^20^ However, when those who make heavy use of cannabis abstain from using it, there is consistent evidence for substitution with alcohol.^8^,^26^,^41^–^43^ Additionally, participants with CUD, compared to those with AUD, report daily patterns of co-use more consistent with substitution.^27^ Motivations for use may be another mechanism by which co-use or cannabis use may impact drinking outcomes. For instance, review of the neurobiological mechanisms suggests that cross-tolerance exists between alcohol and certain cannabinoids. This cross-tolerance may result in increased use among those seeking to experience increased effects from co-use (i.e., “cross-fading”). Alternatively, this cross-tolerance could reinforce substitution motives that might exist, as individuals may experience desired effects from cannabis and be less likely to reach for alcohol in the moment.

Despite several existing gaps, the current literature may shed additional light on these competing theories of substitution versus complementarity. For instance, preliminary work also suggests that individual differences, such as impulsive personality,^117^ may impact drinking rates on co-use days (i.e., less impulsive individuals are more likely to substitute cannabis for alcohol). Additional individual differences that may be factors in whether cannabis acts as a substitute for or a complement to alcohol are important to examine, as both alcohol and cannabis act on the same neural reward pathway; therefore, individual differences in reward sensitivity may interact with co-use to predict unique substitution versus complementary effects. Further, there is a significant dearth of research examining demographic factors—such as sex, race, and ethnicity—that likely play a role in co-use of alcohol and cannabis. For example, evidence from preclinical work suggests that there may be an age-dependent decline in cannabis and alcohol interactions independent from exposure to or level of experience with either substance,^118^ suggesting that age may moderate the level of substitution or complementarity one endorses. Additionally, it should be noted that early studies on cannabis occurred within a criminalized environment, which has led to increased stigmatization of cannabis use^119^ and, therefore, may reduce the ability of those who use cannabis to effectively use it as a substitute for alcohol. Finally, all studies reviewed examined co-use at a single level of analysis (e.g., laboratory administration studies, self-report daily survey studies). However, complex interactions between individual pharmacokinetic response to substance use and an individual’s sociocontextual environment may exist. Mixed-methods studies that cut across these rigorous levels of data collection may help to elucidate how each of these mechanisms (e.g., context of co-use, timing and order of use, cannabinoid formulations, pharmacokinetic interactions, user characteristics) contributes to substitution versus complementary patterns of use. Taken together, these studies highlight the complex nature of cannabis and alcohol co-use and ultimately suggest that internal (e.g., pharmacological) and external (e.g., context) factors interact to yield complementary and substitution effects of alcohol and cannabis that likely shift over time, throughout the day, and potentially in the moment. Further investigation is needed to continue to clarify differences in patterns and context of cannabis use as either a complement to or a substitute for alcohol use at both within- and between-person levels.

## Figures and Tables

**Table 1:** Effects of Substitution or Complementary Use of Cannabis on Use of Alcohol and Alcohol-Related Consequences, by Sample

Substitution	
**Clinical or Treatment-Seeking or -Engaged**	
**Alcohol use**	**Alcohol-related consequences**
Self-reported substitution of ALC with CB^24^	
CB use days associated with lower ALC consumption after ALC Tx^25^	
Decreased CB after contingency management Tx for CUD associated with increased ALC use; reinitiated CB associated with decreased ALC use^26^	
**Non–Treatment-Seeking**	
**Alcohol use**	**Alcohol-related consequences**
CB abstinence associated with increased ALC use^7^,^8^,^41^,^42^	
THC administration associated with increased ALC use and craving^43^	
Combined ALC and CB associated with lower “want more drug”^57^	
**Complementary**	
**Clinical or Treatment-Seeking or -Engaged**	
**Alcohol use**	**Alcohol-related consequences**
CB use after AUD Tx associated with resumed ALC use^16^	More frequent CB use during AUD Tx associated with increased ALC consequences 1 year after Tx^19^
CB at AUD Tx entry associated with reduced abstinence^17^	CB use predicts AUD^14^,^15^
Mid-level CB use frequency during/after AUD Tx associated with fewer abstinent days after Tx, higher quantity, and greater frequency^18^,^20^	
Reduced CB use after CUD Tx associated with reduced ALC use among those with AUD^21^	
Reductions in ALC and CB use among persons in ALC Tx who report heavy drinking and CB use^22^,^23^	
**Non–Treatment-Seeking**	
**Alcohol use**	**Alcohol-related consequences**
Daily CB use associated with more ALC use^27^,^28^	CB use associated with increased ALC consequences^28^,^32^–^34^,^36^
Simultaneous use associated with more ALC use^6^,^10^,^30^	Co-use associated with neurocognitive abnormalities^35^

*Note:* ALC, alcohol; AUD, alcohol use disorder; CB, cannabis; CUD, cannabis use disorder; Tx, treatment
